# Role of Arthrospira Platensis in Preventing and Treating High-Fat Diet-Induced Hypercholesterolemia in Adult Rats

**DOI:** 10.3390/nu16121827

**Published:** 2024-06-11

**Authors:** Nunzio Antonio Cacciola, Paola De Cicco, Maja Milanović, Ivan Milovanović, Aleksandra Mišan, Danijela Kojić, Jelica Simeunović, Dajana Blagojević, Tamara Popović, Aleksandra Arsić, Vladimir Pilija, Anamarija Mandić, Francesca Borrelli, Nataša Milić

**Affiliations:** 1 Department of Veterinary Medicine and Animal Production, University of Naples Federico II, Via F. Delpino 1, 80137 Naples, Italy; nunzioantonio.cacciola@unina.it; 2 Department of Pharmacy, School of Medicine and Surgery, University of Naples Federico II, Via D. Montesano, 49, 80131 Naples, Italy; paola.decicco@unina.it; 3 Department of Pharmacy, Faculty of Medicine, University of Novi Sad, Hajduk Veljkova 3, 21000 Novi Sad, Serbia; maja.milanovic@mf.uns.ac.rs; 4 State Laboratory, Backweston Laboratory Campus, Celbridge, W23 VW2C Co. Kildare, Ireland; ivan.milovanovic@statelab.ie; 5 Institute of Food Technology, University of Novi Sad, Bulevar Cara Lazara 1, 21000 Novi Sad, Serbia; aleksandra.misan@fins.uns.ac.rs (A.M.); anamarija.mandic@fins.uns.ac.rs (A.M.); 6 Department of Biology and Ecology, Faculty of Sciences, University of Novi Sad, Trg Dositeja Obradovića 2, 21000 Novi Sad, Serbia; danijela.kojic@dbe.uns.ac.rs (D.K.); jelica.simeunovic@dbe.uns.ac.rs (J.S.); dajana.blagojevic@dbe.uns.ac.rs (D.B.); 7 Institute for Medical Research, National Institute of Republic of Serbia, University of Belgrade, Dr Subotića starijeg 4, 11129 Belgrade, Serbia; poptam@imi.bg.ac.rs (T.P.); aleksandra.arsic@imi.bg.ac.rs (A.A.); 8 Center for Forensic Medicine, Toxicology and Molecular Genetics, Clinical Centre Vojvodina, Faculty of Medicine, University of Novi Sad, Hajduk Veljkova 3, 21000 Novi Sad, Serbia; vladimir.pilija@mf.uns.ac.rs

**Keywords:** spirulina, hyperlipidaemia, bile acids, hepatoprotective effects, lipid parameters

## Abstract

Hyperlipidaemia is a recognised risk factor for cardiovascular disease. In this study, the antihyperlipidaemic properties of spirulina (Arthrospira platensis, strain S2 from Serbia) were tested in adult Wistar rats before and after induction of hypercholesterolaemia by a high-fat diet (HFD) to compare the preventive with the curative effect. Total cholesterol (TC), high-density lipoprotein cholesterol (HDL-C), low-density lipoprotein cholesterol (LDL-C), alanine transaminase (ALT) and aspartate transaminase (AST) levels were measured in the blood samples. The chemical composition (lipids, proteins and cholesterol) and the content of bile acids in the faeces of the animals were also analysed. Feeding rats with an atherogenic diet for 10 weeks led to the successful development of hyperlipidaemia, as serum TC and LDL-C levels as well as lipids, cholesterol and bile acids in the animals’ faeces were significantly increased. Pre- and post-treatment with spirulina led to a reduction in serum LDL, TC and ALT levels. Administration of spirulina resulted in both a significant increase in primary bile acids excretion and a decrease in bile acids metabolism, with pre-treatment being more effective than post-treatment in some cases. These results suggest that increased excretion of bile acids as well as an effect on the gut microbiota may be the mechanism responsible for the anti-hyperlipidaemic activity of the tested spirulina strain.

## 1. Introduction

Hyperlipidaemia is a chronic condition most commonly associated with the development of cardiovascular disease, one of the leading causes of morbidity and mortality worldwide [1,2]. In 2019, more than 44% and 22% of ischaemic heart disease and stroke deaths worldwide, respectively, were attributable to high plasma LDL/cholesterol levels [3]. Several genetic and environmental factors are associated with an increased risk of hyperlipidaemia. These include a family history of hyperlipidaemia, low physical activity, obesity, smoking, high alcohol consumption and the use of medications [4]. However, the disease is very rare in men and women under the age of 50 [5]. Statins are the drugs of first choice prescribed to lower LDL cholesterol and their use is associated with lower mortality and fewer cardiovascular events [6]. However, in the US, where over 50% of adults have elevated LDL levels, less than 35% of patients treated with statins to lower their levels achieve adequate control [7]. A large-scale randomised clinical trial involving 26 European and non-European countries has also shown that patients respond poorly to treatment, with a median LDL-C reduction of around 50% [8]. In addition, several clinical trials have shown that patient response to statins is highly variable [8], that adherence to treatment is low and that adverse events such as muscle symptoms occur [9,10]. Over the last decade, there has been a great deal of interest in alternative therapies for the treatment of hyperlipidaemia, including the use of herbal medicine.

Spirulina (Arthrospira platensis Gomont also known by the scientific name Spirulina platensis), a blue-green microalgae, is considered one of the most nutritious foods and has been considered the best aliment of the future [11,12,13]. Spirulina has been reported to possess antihyperlipidaemic, antiviral, antibacterial, antioxidant, anti-inflammatory, probiotic, radioprotective, anticancer and immunomodulatory effects [12,13,14]. In addition, several clinical studies have shown the efficacy of spirulina in curing various metabolic diseases by lowering lipid parameters [total cholesterol, triglycerides, low-density lipoprotein cholesterol (LDL-C) and high-density lipoprotein cholesterol (HDL-C)] in hyperlipidaemic, diabetic, obese, non-alcoholic fatty liver and hypertensive patients, while showing no effect in healthy volunteers [15]. So far, no clinical data on a possible preventive effect of spirulina on metabolic diseases and no preclinical studies comparing the preventive and curative effect of spirulina on hyperlipidaemia, especially in adult animals, have been reported. The aim of this study was therefore to evaluate the effect of a spirulina strain of cyanobacteria isolated in Serbia, administered before and after induction of hypercholesterolaemia in adult rats by a high-fat diet, in order to compare the preventive with a curative effect.

## 2. Materials and Methods

### 2.1. Chemicals

Bile acid standards (muricholic acid, hyocholic acid, hyodeoxycholic acid, cholic acid, deoxycholic acid, litocholic acid), cholesterol and formic acid were purchased from Sigma-Aldrich GmbH (Sternheim, Germany). Methanol, acetonitrile, heptane and ethanol of HPLC quality were purchased from Merck (Darmstadt, Germany) and Avantor Performance Materials (Gliwice, Poland). Test kits for total cholesterol (TC), lipoprotein fraction [low-density lipoprotein cholesterol (LDL-C) and high-density lipoprotein cholesterol (HDL-C)], triglycerides (TG), alanine transaminase (ALT) and aspartate transaminase (AST) were obtained from Roche Molecular Diagnostics (Pleasanton, CA, USA). All other reagents and chemicals used were of analytical grade.

### 2.2. Microalgae Biomass Preparation

The tested *Arthrospira platensis* S2 is an autochthonous strain, isolated from Ponjavica River (Serbia) which belongs to the Novi Sad Cyanobacterial Culture Collection (NSCCC) at the Department of Biology and Ecology, Faculty of Sciences, University of Novi Sad. The biomass of *Arthrospira platensis* was prepared in the Department of Biology of the Faculty of Science, University of Novi Sad according to Simeunović [16]. The microalgal biomass was grown in mineral SOT substrate [17] in stationary cultures (in Erlenmeyer flasks), at a temperature of 22–24 °C, in 12 h/12 h light/dark cycles, with a light intensity of 50 µmol/m^2^ s, for 25 days. The spirulina strain was filtered, washed with deionised water, freeze-dried and stored in hermetically sealed containers at +8 °C until use. Regarding the biochemical characterisation, the dry biomass contains 52.7% of the total proteins and 8.9% of the total lipids, the main fatty acids being palmitic acid, γ-linolenic acid and linoleic acid. The total carotenoid content was found to be 10.3 mg/g, while the chlorophyll a, phycocyanin and allophycocyanin content was 31.3 µg/mL, 18.53 µg/mL and 26.34 µg/mL, respectively.

### 2.3. Animals and Experimental Design

All in vivo experiments and protocols were approved by the Committee for the Use and Keeping of Laboratory Animals of the University of Novi Sad (No. III-2011-01). All necessary procedures were used to reduce the suffering of the rats. Forty adult male Wistar rats (4 months old) were obtained from the vivarium of the pharmaceutical company Galenika a.d., Belgrade, Serbia.

The animals were housed in metal cages at room temperature (20 ± 5 °C) with a 12 h/12 h light/dark cycle and free access to water and standard pelleted food. The complete feed with a protein content of 20% was obtained from the Veterinary Institute Subotica, Serbia. Before the start of the experiment, the animals were left free to acclimatise for 14 days and fed with standard pelleted feed. The animals were then randomly divided into five groups (eight rats in each group) as follows:

- Group I (control group), fed with a standard diet for laboratory rats.
- Group II, fed with a standard diet and treated with the biomass of the spirulina strain.
- Group III (atherogenic control group), fed with a high-fat diet (HFD), standard diet supplemented with an atherogenic mixture consisting of 20% sunflower oil, 2.5% cholesterol and 0.5% cholic acid.
- Group IV, fed with a HFD (like group III) and treated with a spirulina biomass strain (preventive treatment).
- Group V, fed with a HFD (like group III) and, after the development of hyperlipidaemia in the fifth week, treated with Spirulina biomass (curative treatment).

Spirulina was administered daily (groups II, IV and V) by oral gavage at a dose of 650 mg/kg (suspended in 1 mL water) during the 5-week (curative protocol) or 10-week (preventive protocol) experimental period. The spirulina dose was selected based on clinical studies performed in elderly patients in which a dose of 7.5 g/die was used [18] and calculated by the body surface area normalisation method [19]. Body weight was measured weekly. The faeces were collected at the end of the experimental period, packed in plastic boxes and stored at −20 °C until analysis. At the end of the experiment, blood was collected from each animal (by cardiac puncture) and transferred to eppendorfs containing sodium citrate as an anticoagulant (3.8%, *w*/*v*). The plasma samples were centrifuged (3000 rpm, 10 min) and stored at −20 °C until use.

### 2.4. Biochemical Analyses and Parameters for Atherosclerosis

The C-111 biochemical analyser (Roche Diagnostic, Sees, France) was used to perform the biochemical analyses of the plasma. TC, HDL-C, LDL-C, ALT and AST concentrations were determined by automated enzymatic methods according to the manufacturer’s instructions. The atherogenicity coefficient (AC: TC-HDL-C)/(HDL-C) and the cardiovascular risk ratio (CRR: TC/HDL-C) were calculated for each group (I–V).

### 2.5. High Performance Liquid Chromatography (HPLC) Determination of Cholesterol in Faeces

Sample preparation and analysis of cholesterol content were performed by liquid Sample preparation and analysis of cholesterol content were performed by liquid chromatography according to the modified method of Oh and colleagues [20]. For direct saponification, the faecal sample was homogenised (using a laboratory mortar and pestle, with impurities such as sawdust residues and excess hair removed). Approximately 0.5 g of the sample was weighed (glass vial with Teflon cap) and 0.5 mL KOH solution (50% in water, *w*/*w*) and 2.5 mL ethanol (96%, *v*/*v*) were added. The samples were saponified at 70 °C for 30 min. The tubes were then cooled to room temperature, centrifuged (2000 rpm, 5 min) and the supernatants were extracted with hexane (3 × 3 mL). The hexane layers were washed with distilled water (3 × 30 mL) and the solvent was evaporated (under the nitrogen stream). The residue was redissolved in 1 mL HPLC ethanol. This solution was diluted 50 times with HPLC ethanol, filtered through a syringe philtre with a pore diameter of 0.22 μm (PTFE, Rotilabo-Spritzenfilter 13 mm, Roth, Karlsruhe, Germany) and transferred to a vial for HPLC analysis.

The cholesterol content was determined by liquid chromatography on HPLC Agilent 1200 series with diode array detector (DAD) at 212 nm according to the modified method of Oh and colleagues [20] using a Zorbax Eclipse Plus C18 (1.8 μm, 4.6 mm × 50 mm) column. Methanol was used as the mobile phase (flow rate 1.0 mL/min). The temperature of the column was 30 °C and the injection volume was 10 μL using the autosampler. The cholesterol standard was dissolved in HPLC ethanol in a concentration range of 0.01 to 1.00 mg/mL. The cholesterol concentration was calculated from the curve by linear regression and expressed in mg/g of faecal sample.

### 2.6. HPLC Determination of Bile Acids in Faeces

The extraction of bile acids from faeces was performed according to the modified method of Sjövall and colleagues [21]. Homogenised faecal samples were extracted with ethanol (80%, *v*/*v*, 3 × 2 mL, 100 °C, 15 min) and the extracts were subsequently evaporated under a stream of nitrogen. The dried extracts were resuspended in 0.6 mL methanol and 2.4 mL distilled water. The extract was purified by solid phase extraction (SPE, OPT SPE 60 mg cartridge, Agilent Technologies, Santa Clara, CA, USA). The SPE column was conditioned with 3 mL methanol and 3 mL water (flow rate 1 mL/min). After the prepared extracts were passed through the column, they were washed with 1 mL of a methanol-in-water solution (5%, *v*/*v*) and eluted with 2 mL of methanol. The eluted solvent was then evaporated under a stream of nitrogen and the dried residue was reconstituted in 1 mL of the mobile phase, filtered through a syringe philtre with 0.22 μm pore diameter (PTFE; Rotilabo-Spritzenfilter 13 mm, Roth, Karlsruhe, Germany) and transferred to a vial. Bile acids were determined in 10 μL of the prepared faecal extract by the liquid chromatography method (Agilent 1200 HPLC series with Zorbax Eclipse Plus C18 column, 1.8 μm, 4.6 mm × 50 mm; 30 °C) using the evaporative light scattering detector (ELSD). The detector parameters were: 3.5 bar carrier gas pressure (nitrogen), temperature 40 °C, gain value 1. A mixture of methanol and formic acid solution in water (1%, *v*/*v*, ratio 75:25) with a flow rate of 1.0 mL/min was used as the mobile phase. The bile acid standards (muricholic acid, hyocholic acid, hyodeoxycholic acid, cholic acid, deoxycholic acid, litocholic acid) were dissolved in the mobile phase and a dilution series was prepared for each acid in the concentration range of 0.01–1 mg/mL. The bile acids concentration was calculated using the linear regression equations obtained and expressed in mg/g faecal sample.

### 2.7. Total Calorific Value of Faeces

The total calorific value of faeces was determined using the AC500 calorimeter (Leco Corporation, Plzen, Czech Republic). The faecal samples were homogenised (using a mortar and pestle) and formed into solid pellets using an automatic tableting machine. Calibration of the calorimeter was performed with the standard benzoic acid (99.2%, Leco Corporation, Plzen, Czech Republic). The results are given as MJ/kg.

### 2.8. Determination of Proteins and Lipids in the Faeces

The total protein content in faeces was determined according to the official AOAC method, number 992.23 [22] using the TruSpec device (Leco corporation, Plzen, Czech Republic) whereas the Soxhlet extraction method with petroleum ether was applied for the estimation of lipids in the faeces [23].

### 2.9. Statistical Analysis

Results are expressed as mean ± SEM of 6/8 mice for each experimental group. Group comparisons were assessed using one-way ANOVA (followed by Tukey’s multiple comparison test). Analyses were performed using GraphPad Prism 9.2.0 (La Jolla, CA, USA). A *p* value of less than 0.05 was considered significant.

## 3. Results and Discussion

### 3.1. Changes of Body Weight

**Figure 1:**
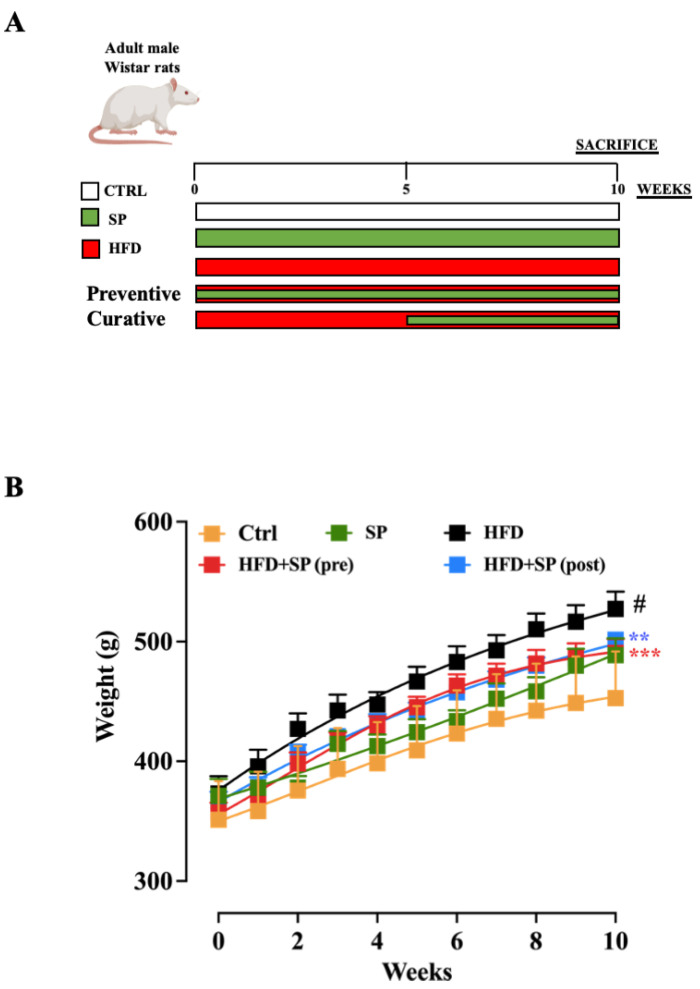
(**A**) Experimental design related to the animal model of HFD-induced hyperlipidaemia. (**B**) Weight changes in mice fed standard chow diet (ctrl), spirulina diet (SP), high fat diet (HFD), HFD + SP (pre-treatment) and HFD + SP (post-treatment). Spirulina was administered daily by oral gavage at a dose of 650 mg/kg (suspended in 1 mL water) during the 5-week (curative protocol, i.e., post-treatment) or 10-week (preventive protocol, pre-treatment) experimental period. Body weight was measured weekly for 10 weeks. Data represent the mean ± SEM of 8 mice per group. Two-way ANOVA followed by Tukey’s multiple comparisons test was performed to test for differences among the groups. # *p* < 0.0001 vs. ctrl group; ** *p* < 0.01 and *** *p* < 0.001 vs. HFD group.

Considering that hyperlipidaemia is a disease that mainly affects people over 50, adult male Wistar rats (4 months old) were used in this study. At the beginning of the study, the rats in all five groups had a similar average initial body weight. After 10 weeks of the experimental period (experimental design reported in Figure 1A), the body weight of the rats fed the control or HFD diet increased significantly, with the body weight in the HFD group being significantly higher than that of the control group (Figure 1B). These results are consistent with other studies in which the HFD significantly increased the body weight of rodents [24]. Administration of spirulina before and after the induction of hyperlipidaemia significantly reduced the increase in body weight (Figure 1B), with no significant differences between pre- and post-treatment.

### 3.2. Hyperlipidaemia and Hepatic Biochemical Markers

**Figure 2:**
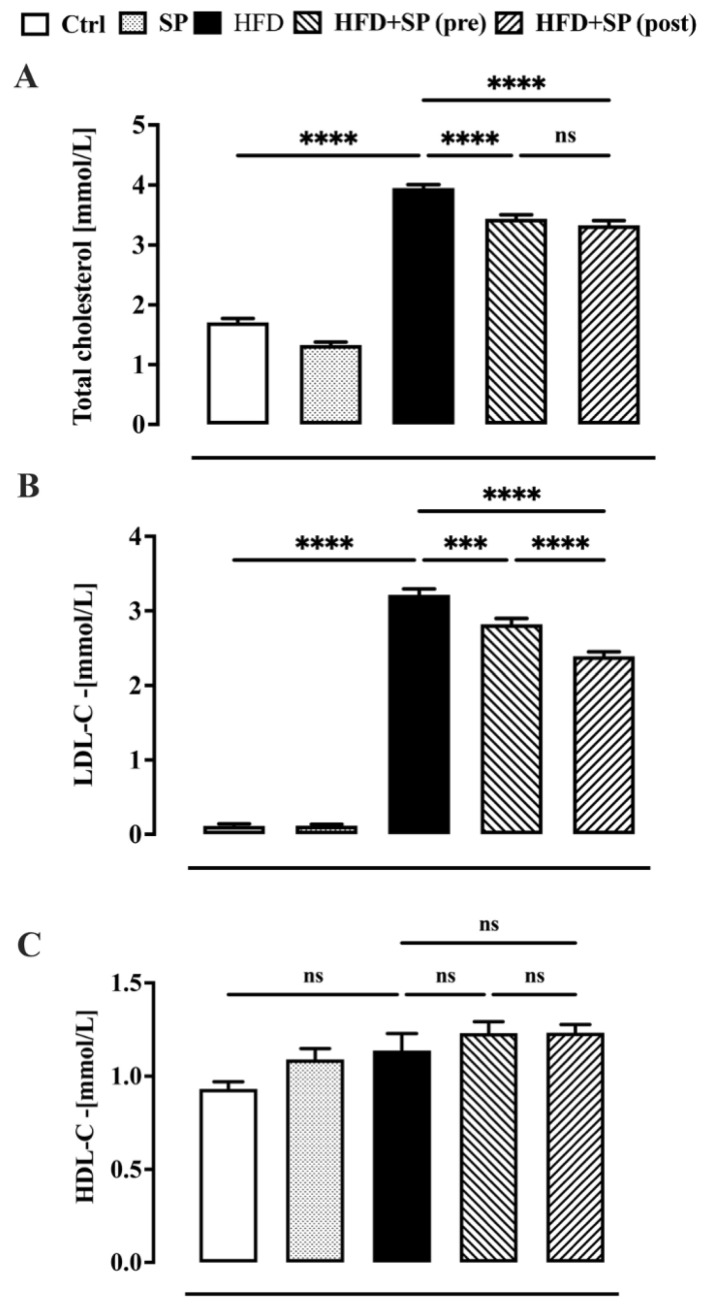
Plasma lipid profile in mice fed standard chow diet (ctrl), spirulina diet (SP), high fat diet (HFD), HFD + SP (pre-treatment) and HFD + SP (post-treatment). Spirulina was administered daily by oral gavage at a dose of 650 mg/kg (suspended in 1 mL water) during the 5-week (curative protocol, i.e., post-treatment) or 10-week (preventive protocol, pre-treatment) experimental period. (**A**) total cholesterol, (**B**) LDL-cholesterol and (**C**) HDL-cholesterol. Bars represent the mean ± SEM of 8 mice per group. Ordinary one-way ANOVA with Tukey’s post hoc analysis was used to evaluate differences between experimental groups. *** *p* < 0.001 and **** *p* < 0.0001; ns = no significant.

Dyslipidaemia is associated with altered levels of TC, LDL-C and HDL-C levels which enhance the risk of cardiovascular disease (CVD) [1]. According to the literature [25], our study found that all lipid parameters, except HDL-C, were significantly higher in the animals fed a high-fat diet (HDF) than in the control group (i.e., animals fed a standard food) (Figure 2). Administration of spirulina before and after the development of hyperlipidaemia significantly reduced the increase in TC and LDL-C induced by the HFD (Figure 2A,B). The curative effect of spirulina on LDL-C levels was significantly more pronounced than the preventive effect (Figure 2B). Moreover, spirulina administered to healthy animals had no significant effect on lipid levels. The results obtained suggest that the anti-hyperlipidaemic effect of spirulina was more pronounced when this microalga was added to the diet when hyperlipidaemia was already present. The current findings are consistent with previous results showing that dietary supplementation with natural anti-hyperlipidaemic substances had a more pronounced effect in hyperlipidaemic subjects than in subjects with normal lipid status [26]. Other studies have reported the positive effect of spirulina on lipid parameters, but no one has compared the preventive effect with the curative effect [27,28].

Atherosclerosis parameters have been described as meaningful indicators of CVD risk; the higher the score, the higher the risk of developing the disease and viceversa [29]. In this study, similar to lipid parameters, atherosclerosis parameters such as atherosclerosis coefficient (AC) and cardiovascular risk ratio (CRR), which were dramatically increased in rats receiving HFD, were reduced by spirulina (SP) supplementation (Table 1). The significant reduction in AC (31.2% and 35.2% before and after spirulina treatment, respectively) and CRR (23.7% and 25.6% before and after spirulina treatment, respectively) confirmed the cardioprotective effect of spirulina. 

**Figure 3:**
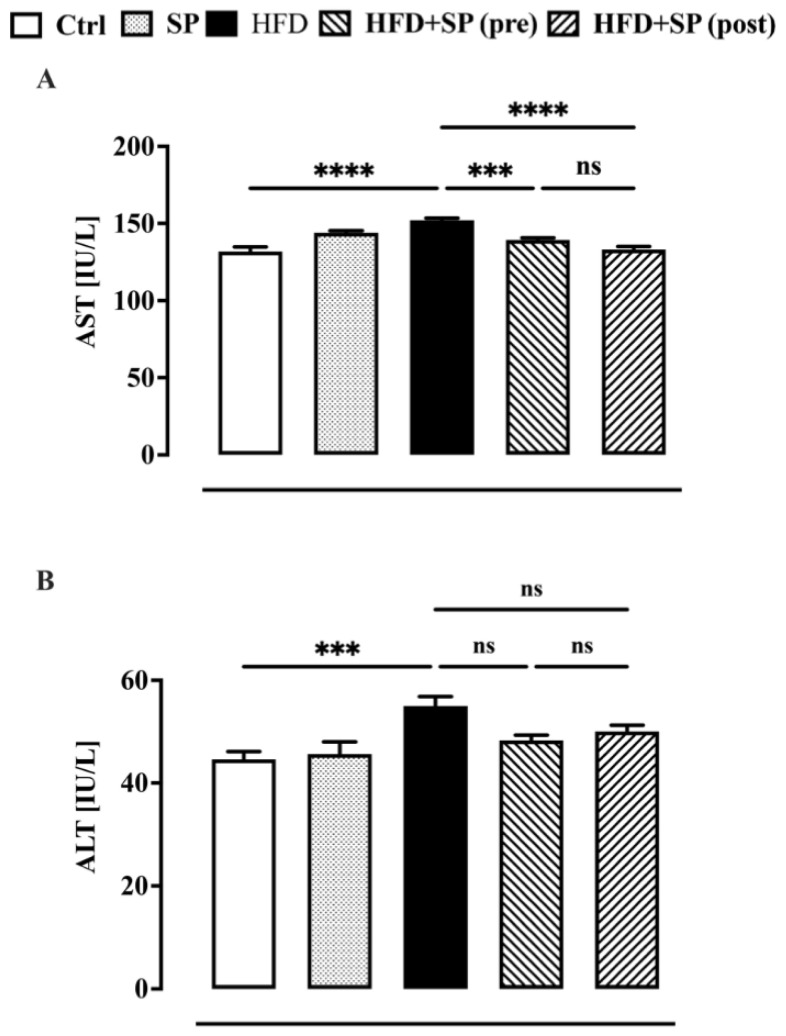
Liver function biomarkers measured in the plasma of mice fed standard chow diet (ctrl), spirulina diet (SP), high fat diet (HFD), HFD + SP (pre-treatment) and HFD + SP (post-treatment). Spirulina was administered daily by oral gavage at a dose of 650 mg/kg (suspended in 1 mL water) during the 5-week (curative protocol, i.e., post-treatment) or 10-week (preventive protocol, pre-treatment) experimental period. (**A**) aspartate aminotransferase (AST) and (**B**) alanine aminotransferase (ALT). Bars represent the mean ± SEM of 8 mice per group. Ordinary one-way ANOVA with Tukey’s post hoc analysis was used to evaluate differences between experimental groups. *** *p* < 0.001 and **** *p* < 0.0001; ns = no significant.

In the last ten years, a correlation between an elevated lipid profile and liver enzymes has been demonstrated [30,31]. Therefore, in this work, we also investigated the effect of spirulina on the two major liver enzymes (i.e., ALT and AST), which are considered indicators of fatty liver infiltration due to lipid status disorders [32]. In agreement with the literature [33], the animals fed a high-fat diet showed higher AST and ALT levels compared to the control group (Figure 3). Spirulina supplementation administered before and after the development of hyperlipidaemia was able to significantly reduce the HFD-induced increase in AST but not ALT (Figure 3). Our results are in contrast to other papers reporting a beneficial effect of spirulina on AST and ALT, which are altered by HFD and other animal models of liver disease. The discrepancy between our results and other papers could be due to several factors, including a different HFD, the age of the animals, and the dosage and composition of spirulina [34].

### 3.3. Chemical Composition, Cholesterol and Bile Acid Content of Faeces

It has been suggested that the observed anti-hyperlipidaemic effect of spirulina is partly related to changes in cholesterol metabolism and absorption [35,36]. To elucidate the mechanism of the observed anti-hyperlipidaemic activity of the tested spirulina strain, the collected faecal samples were analysed to determine the levels of proteins, lipids, cholesterol and total energy. As we expected, the energy value, protein, lipid and cholesterol levels in the faeces of the animals fed the atherogenic diet differed significantly from those of the control group (Table 2). 

In particular, consumption of the HFD resulted in a significant increase in total caloric value, lipids and cholesterol and a decrease in protein levels in the faeces of the animals (Table 2). Spirulina treatment (both pre- and post-treatment) inhibited the reduction in protein levels but had no effect on the HFD-induced increase in cholesterol levels, suggesting that cholesterol metabolism and absorption are not a major target for the hypocholesterolaemic effect of the tested spirulina strain (Table 2). However, post-treatment with spirulina, but not pre-treatment, led to a further increase in the amount of lipid excreted in the faeces (Table 2), confirming that natural compounds with antihyperlipidaemic activity act preferentially on hyperlipidaemic subjects than on healthy subjects [26]. Another possible mechanism that is suspected for the biomolecules of cyanobacteria is the direct influence on the absorption of bile acids in the intestine. It is known that bile acids are synthesised from cholesterol in the liver and released into the duodenum, where they emulsify the absorbed fats and aid digestion [37,38]. Subsequently, more than 90% of the bile acids are absorbed from the small intestine. The remainder is metabolised in the large intestine and then partially reabsorbed [37,38]. The amount of bile acids excreted in the faeces is therefore very modest. Humans synthesise cholic acid (CA) and chenodeoxycholic acid as primary bile acids, while mice synthesise CA and β-muricholic acid (βMCA) [39,40]. Increased faecal excretion of bile acids (e.g., using resins that sequester bile acids) stimulates ex novo synthesis of the same at the hepatic level, which determines the consumption of cholesterol withdrawn from the liver and plasma of the depot [41,42]. To meet the increased demand, the liver increases the expression of its receptors for LDL (bad cholesterol), which lowers total and LDL cholesterol levels [32,33]. To determine the effect of spirulina on the enterohepatic circulation of bile acids, in this work the content of the main primary and secondary bile acids (i.e., βMCA, CA, hyocholic acid, hyodeoxycholic acid, deoxycholic acid and lithocholic acid) in faeces was determined. Our experiments showed that the levels of βMCA, CA, hyocholic acid, hyodeoxycholic acid, deoxycholic acid and lithocholic acids were significantly increased in the faeces of animals fed atherogenic diets compared to the control group (Table 3). 

Pretreatment with spirulina resulted in both a significant increase in the levels of the primary bile acid cholic acid and a decrease in the levels of its metabolite deoxycholic acid in the faeces of the HFD-fed animals (Table 3). These results suggest that the addition of spirulina to an atherogenic diet led to a reduction in (i) the absorption of cholic acid and, in turn, a reduction in plasma cholesterol and LDL levels and (ii) the conversion of cholic acid to its metabolite deoxycholic acid, probably through an effect on the gut microbiota. A possible effect of spirulina on the gut microbiota also seems to be confirmed by the significant reduction in the levels of another secondary metabolite, lithocholic acid (Table 3). The levels of βMCA, hyocholic acid and hyodeoxycholic acid were not altered by spirulina pretreatment. Similarly, spirulina administered after the induction of hyperlipidaemia was able to reduce both the absorption of CA and the conversion of CA to deoxycholic acid, albeit with less efficacy (Table 3). In contrast to the preventive treatment, the post-treatment with spirulina was also able to increase the level of βMCA. Interestingly, pre-treatment with spirulina was not able to alter the content of hyodeoxycholic acid, a secondary bile acid produced by intestinal bacteria from the isomers of muricholic acids and hyocholic acid [43], while post-treatment with spirulina significantly increased this secondary bile acid, suggesting a different mechanism of action of pre- and post-treatment with spirulina. 

## 4. Conclusions

In summary, all these data seem to indicate that the increased excretion of bile acids could be the mechanism responsible for the anti-hyperlipidaemic activity of the tested spirulina strain. In addition, the increased excretion of cholic acid in unchanged form observed in this experiment and thus its reduced conversion into secondary bile acids could indicate the influence of some biomolecules of the cyanobacteria of the Spirulina genus on bile acid metabolism through an effect on the gut microbiota. Further research in this direction is required to explain these observed effects.

## Figures and Tables

**Table 1:** Atherosclerosis parameters in rats receiving high fat diet (HFD) and/or spirulina.

Group	Atherogenicity Coefficient (AC)	Cardiovascular Risk Ratio (CRR) (%, *w*/*w*)
I	0.85 ± 0.09	1.86 ± 0.09
II	0.25 ± 0.09	1.23 ± 0.09
III	2.66 ± 0.36 #	3.67 ± 0.36 #
IV	1.83 ± 0.13 *	2.83 ± 0.13 *
V	1.73 ± 0.13 *	2.73 ± 0.13 *

Group I, fed with a standard diet; Group II, fed with a standard diet and treated with spirulina; Group III, fed with a high-fat diet (HFD); Group IV, fed with a HFD and treated with spirulina (preventive treatment); Group V, fed with a HFD and, after the development of hyperlipidaemia in the fifth week, treated with Spirulina (curative treatment). Values are expressed as Mean ± SEM; # *p* < 0.0001 vs. control (group I); * *p* < 0.05 vs. HFD (group III), *n* = 8 samples.

**Table 2:** Total caloric value, protein, lipids and cholesterol in faeces.

Group	Total Caloric Value (MJ/kg)	Protein (%, *w*/*w*)	Lipids (%, *w*/*w*)	Cholesterol (mg/g)
I	14.6 ± 0.04	19.7 ± 0.10	1.90 ± 0.07	0.43 ± 0.02
II	14.7 ± 0.04	22.6 ± 0.07	2.38 ± 0.03	0.50 ± 0.02
III	18.7 ± 0.08 #	15.5 ± 0.17 #	11.6 ± 0.04 #	14.7 ± 0.42 #
IV	18.5 ± 0.05	16.6 ± 0.01 ****	11.5 ± 0.01	15.9 ± 0.51
V	19.2 ± 0.03 ****	17.4 ± 0.04 ****	13.2 ± 0.04 ****	13.6 ± 0.06

Group I, fed with a standard diet; Group II, fed with a standard diet and treated with spirulina; Group III, fed with a high-fat diet (HFD); Group IV, fed with a HFD and treated with spirulina (preventive treatment); Group V, fed with a HFD and, after the development of hyperlipidaemia in the fifth week, treated with Spirulina (curative treatment). Values are expressed as Mean ± SEM; # *p* < 0.0001 vs. control (group I); **** *p* < 0.0001 vs. HFD (group III), *n* = 8 samples.

**Table 3:** The content of bile acids in faeces of experimental groups of laboratory rats.

	Content of Bile Acids (mg/g) in Different Animal Groups				
Bile Acid	I	II	III	IV	V
β-Muricholic	0.82 ± 0.01	0.79 ± 0.02	4.32 ± 0.09 #	3.96 ± 0.05	4.70 ± 0.17 *
Cholic	0.19 ± 0.01	0.28 ± 0.01	1.23 ± 0.04 #	2.24 ± 0.19 ****	1.91 ± 0.08 ***
Hyocholic	0.92 ± 0.01	0.78 ± 0.01	0.66 ± 0.04 #	0.60 ± 0.01	0.63 ± 0.01
Hyodeoxycholic	1.22 ± 0.01	0.96 ± 0.01	1.87 ± 0.12 #	2.01 ± 0.03	2.17 ± 0.08 *
Deoxycholic	0.66 ± 0.01	0.53 ± 0.03	13.04 ± 0.61 #	9.63 ± 0.50 ****	11.4 ± 0.36 *
Litocholic	0.56 ± 0.01	0.54 ± 0.03	1.20 ± 0.03 #	0.81 ± 0.02 ****	1.07 ± 0.03 **

Group I, fed with a standard diet; Group II, fed with a standard diet and treated with spirulina; Group III, fed with a high-fat diet (HFD); Group IV, fed with a HFD and treated with spirulina (preventive treatment); Group V, fed with a HFD and, after the development of hyperlipidaemia in the fifth week, treated with Spirulina (curative treatment). Values are expressed as Mean ± SEM; # *p* < 0.0001 vs. control (group I); * *p* < 0.05, ** *p* < 0.01, *** *p* < 0.001 and **** *p* < 0.0001 vs. HFD (group III), *n* = 8 samples.

## Data Availability

The original contributions presented in the study are included in the article further inquiries can be directed to the corresponding authors.

## References

[1] Gaggini M., Gorini F., Vassalle C. (2022). Lipids in Atherosclerosis: Pathophysiology and the Role of Calculated Lipid Indices in Assessing Cardiovascular Risk in Patients with Hyperlipidemia. Int. J. Mol. Sci..

[2] Roth G.A., Mensah G.A., Johnson C.O., Addolorato G., Ammirati E., Baddour L.M., Barengo N.C., Beaton A.Z., Benjamin E.J., Benziger C.P. (2020). Global Burden of Cardiovascular Disease and Risk Collaborators, 1990–2019: Update from the GBD 2019 Study. J. Am. Coll. Cardiol..

[3] Global Health Data Exchange (2021). GBD Results Tool. Institute for Health Metrics and Evaluation. http://ghdx.healthdata.org/gbd-results-tool.

[4] Mensah G.A., Fuster V., Murray C.J.L., Roth G.A., Global Burden of Cardiovascular D., Risks C. (2023). Global Burden of Cardiovascular Diseases and Risks, 1990–2022. J. Am. Coll. Cardiol..

[5] Rosada A., Kassner U., Weidemann F., Konig M., Buchmann N., Steinhagen-Thiessen E., Spira D. (2020). Hyperlipidemias in elderly patients: Results from the Berlin Aging Study II (BASEII), a cross-sectional study. Lipids Health Dis..

[6] Grundy S.M., Stone N.J., Bailey A.L., Beam C., Birtcher K.K., Blumenthal R.S., Braun L.T., de Ferranti S., Faiella-Tommasino J., Forman D.E. (2019). 2018 AHA/ACC/AACVPR/AAPA/ABC/ACPM/ADA/AGS/APhA/ASPC/NLA/PCNA Guideline on the Management of Blood Cholesterol: Executive Summary: A Report of the American College of Cardiology/American Heart Association Task Force on Clinical Practice Guidelines. J. Am. Coll. Cardiol..

[7] Karr S. (2017). Epidemiology and management of hyperlipidemia. Am. J. Manag. Care.

[8] Ridker P.M., Mora S., Rose L., Group J.T.S. (2016). Percent reduction in LDL cholesterol following high-intensity statin therapy: Potential implications for guidelines and for the prescription of emerging lipid-lowering agents. Eur. Heart J..

[9] Liu T., Zhao D., Qi Y. (2022). Global Trends in the Epidemiology and Management of Dyslipidemia. J. Clin. Med..

[10] Newman C.B., Preiss D., Tobert J.A., Jacobson T.A., Page R.L., Goldstein L.B., Chin C., Tannock L.R., Miller M., Raghuveer G. (2019). Statin Safety and Associated Adverse Events: A Scientific Statement from the American Heart Association. Arterioscler. Thromb. Vasc. Biol..

[11] Gentscheva G., Nikolova K., Panayotova V., Peycheva K., Makedonski L., Slavov P., Radusheva P., Petrova P., Yotkovska I. (2023). Application of Arthrospira platensis for Medicinal Purposes and the Food Industry: A Review of the Literature. Life.

[12] Jung F., Krüuger-Genge A., Waldeck P., Küpper J.-H. (2019). Spirulina platensis, a Super Food?. J. Cell. Biotechnol..

[13] Banakar V., Alam Q., Rajendra S.V., Pandit A., Cladious A., Gnanaprakash K. (2020). Spirulina, The Boon of Nature. Int. J. Res. Pharm. Sci..

[14] Maddiboyina B., Vanamamalai H.K., Roy H., Ramaiah, Gandhi S., Kavisri M., Moovendhan M. (2023). Food and drug industry applications of microalgae Spirulina platensis: A review. J. Basic Microbiol..

[15] Rahnama I., Arabi S.M., Chambari M., Bahrami L.S., Hadi V., Mirghazanfari S.M., Rizzo M., Hadi S., Sahebkar A. (2023). The effect of Spirulina supplementation on lipid profile: GRADE-assessed systematic review and dose-response meta-analysis of data from randomized controlled trials. Pharmacol. Res..

[16] Simeunović J. (2005). Kolekcija Kultura Cijanobakterija.

[17] Soong P., Shelef G., Soeder C.J. (1980). Production and development of Chlorella and Spirulina in Taiwan. Algae Biomass: Production and Use.

[18] Deng R., Chow T.J. (2010). Hypolipidemic, antioxidant, and antiinflammatory activities of microalgae Spirulina. Cardiovasc. Ther..

[19] Sharma V., McNeill J.H. (2009). To scale or not to scale: The principles of dose extrapolation. Br. J. Pharmacol..

[20] Oh H.I., Shin T.S., Chang E.J. (2001). Determination of Cholesterol in Milk and Dairy Products by High-Performance Liquid Chromatography. Asian-Australas. J. Anim. Sci..

[21] Sjövall J., Griffiths W.J., Setchell K.D.R., Mano N., Goto J., Makin H., Gower D. (2010). Analysis of Bile Acids. Steroid Analysis.

[22] AOAC (2000). Official Methods of Analysis.

[23] AOAC (1984). Official Methods of Analysis.

[24] Fellmann L., Nascimento A.R., Tibiriça E., Bousquet P. (2013). Murine models for pharmacological studies of the metabolic syndrome. Pharmacol. Ther..

[25] Jia Y.J., Liu J., Guo Y.L., Xu R.X., Sun J., Li J.J. (2013). Dyslipidemia in rat fed with high-fat diet is not associated with PCSK9-LDL-receptor pathway but ageing. J. Geriatr. Cardiol..

[26] Lairon D. (1996). Dietary fibres: Effects on lipid metabolism and mechanisms of action. Eur. J. Clin. Nutr..

[27] Devi M.A., Venkataraman L.V. (1983). Hypocholesterolemic effect of blue-green algae Spirulina platensis in albino rats. Ann. Nutr. Rep. Int..

[28] Kato T., Takemoto K., Katayama H., Kuwabara Y. (1984). Effects of Spirulina (Spirulina platensis) on dietary hypercholesterolemia in rats. J. Jpn. Soc. Nutr. Food Sci..

[29] Dobiášová M. (2004). Atherogenic Index of Plasma [Log(Triglycerides/HDL-Cholesterol)]: Theoretical and Practical Implications. Clin. Chem..

[30] Kathak R.R., Sumon A.H., Molla N.H., Hasan M., Miah R., Tuba H.R., Habib A., Ali N. (2022). The association between elevated lipid profile and liver enzymes: A study on Bangladeshi adults. Sci. Rep..

[31] Deb S., Puthanveetil P., Sakharkar P. (2018). A Population-Based Cross-Sectional Study of the Association between Liver Enzymes and Lipid Levels. Int. J. Hepatol..

[32] Sharma R., Watson R.R., Preedy V.R. (2013). Biochemical mechanisms of fatty liver and bioactive foods: Fatty liver, diagnosis, nutrition therapy. Bioactive Food as Dietary Interventions for Liver and Gastrointestinal Disease.

[33] Lasker S., Rahman M.M., Parvez F., Zamila M., Miah P., Nahar K., Kabir F., Sharmin S.B., Subhan N., Ahsan G.U. (2019). High-fat diet-induced metabolic syndrome and oxidative stress in obese rats are ameliorated by yogurt supplementation. Sci. Rep..

[34] Mazokopakis E.E., Papadomanolaki M.G., Fousteris A.A., Kotsiris D.A., Lampadakis I.M., Ganotakis E.S. (2014). The hepatoprotective and hypolipidemic effects of Spirulina (*Arthrospira platensis*) supplementation in a Cretan population with non-alcoholic fatty liver disease: A prospective pilot study. Ann. Gastroenterol..

[35] Colla L.M., Muccillo-Baisch A.L., Costa J.A.V. (2008). Spirulina platensis effects on the levels of total cholesterol, HDL and triacylglycerols in rabbits fed with a hypercholesterolemic diet. Braz. Arch. Biol. Tech..

[36] Nagaoka S., Shimizu K., Kaneko H., Shibayama F., Morikawa K., Kanamaru Y., Otsuka A., Hirahashi T., Kato T. (2005). A novel protein C-phycocyanin plays a crucial role in the hypocholesterolemic action of Spirulina platensis concentrate in rats. J. Nutr..

[37] Chiang J.Y. (2013). Bile acid metabolism and signaling. Compr. Physiol..

[38] Guzior D.V., Quinn R.A. (2021). Review: Microbial transformations of human bile acids. Microbiome.

[39] Guo G.L., Chiang J.Y.L. (2020). Is CYP2C70 the key to new mouse models to understand bile acids in humans?. J. Lipid Res..

[40] Li J., Dawson P.A. (2019). Animal models to study bile acid metabolism. Biochim. Biophys. Acta Mol. Basis Dis..

[41] Feingold K.R., Feingold K.R., Anawalt B., Blackman M.R., Boyce A., Chrousos G., Corpas E., de Herder W.W., Dhatariya K., Dungan K., Hofland J. (2000). Cholesterol Lowering Drugs. Endotext.

[42] Stanciu M.C., Nichifor M., Teaca C.A. (2023). Bile Acid Sequestrants Based on Natural and Synthetic Gels. Gels.

[43] Eyssen H.J., De Pauw G., Van Eldere J. (1999). Formation of hyodeoxycholic acid from muricholic acid and hyocholic acid by an unidentified gram-positive rod termed HDCA-1 isolated from rat intestinal microflora. Appl. Environ. Microbiol..

